# Enhanced power density in solid oxide fuel cells using nickel-assisted gadolinium-doped ceria anodes

**DOI:** 10.1371/journal.pone.0326559

**Published:** 2025-06-27

**Authors:** Nacer Badi, Aashis S. Roy, Raghavendra Sagar, Saleh A. Alghamdi, Abdulrhman M. Alsharari, Alex Ignatiev

**Affiliations:** 1 Thermal Management and Sustainability Research Laboratory, Department of Physics, Faculty of Science, University of Tabuk, Tabuk, Saudi Arabia; 2 Renewable Energy & Environmental Technologies Research Center, University of Tabuk, Tabuk, Saudi Arabia; 3 Department of Chemistry, S.S. Tegnoor Degree College, Kalaburagi, Karnataka, India; 4 Department of Physics, Mangalore Institute of Technology & Engineering, Badaga Mijar, Moodbidri, Karnataka, India; 5 Department of Physics, Faculty of Science, University of Tabuk, Tabuk, Saudi Arabia; 6 Department of Physics, University of Houston, Houston, Texas, United States of America; Indian Institute of Technology Kharagpur, INDIA

## Abstract

This study demonstrates the use of Gadolinium-doped ceria (GDC) (Ce₀.Gd₀.₂O₂) as the anode, BaNb₄MoO₂₀ (BNMO) as the electrolyte, and Lanthanum strontium cobalt oxide (LSCO) (La₀.Sr₀.₄CoO₃) as the cathode in the fabrication of a solid oxide fuel cell (SOFC). The synthesized nanocomposites were characterized using Fourier-transform infrared spectroscopy (FTIR) and X-ray diffraction (XRD) for structural analysis, and scanning electron microscopy (SEM) for surface morphology assessment. DC conductivity measurements revealed that LSCO exhibited a high conductivity of 5.2 S/cm, attributed to the efficient flow of electrons through the electrolyte, highlighting its potential as a promising cathode material. Nyquist plots displayed semi-circular arcs, which correspond to distinct electrochemical processes within the system. The diameter of these arcs reflects the charge transfer resistance, primarily due to grain boundary resistance, while the initial resistance preceding the arc is associated with the bulk properties of the electrolyte. Beyond the first semicircle, diffusion resistance increases with frequency as a result of electrode polarization. It was also observed that the cell voltage dropped in discrete steps when the current density reached 200 mA/cm^2^. Specifically, the voltage decreased from 0.75 V to 0.53 V at 500°C, and from 0.98 V to 0.73 V at 800°C, likely due to charge transfer resistance at the electrode-electrolyte interface. The power density curve indicated that the cell achieved power densities of approximately 0.094, 0.118, 0.146, and 0.184 W/cm^2^ at operating temperatures of 500, 600, 700, and 800°C, respectively, demonstrating favorable performance for an SOFC employing BNMO as the electrolyte.

## Introduction

Ceria-doped gadolinium oxide (CGO), commonly referred to as Gadolinium-doped ceria (Ce₀.Gd₀.₂O₂), has garnered significant attention as a potential anode material in Solid Oxide Fuel Cells (SOFCs). CGO exhibits unique electrical, thermal, and electrochemical properties that contribute to the high stability and superior performance of SOFCs [1,2]. It offers an ionic conductivity of approximately 0.104 S·cm⁻^1^ at 700°C, attributed to the high mobility of oxygen ions, coupled with an activation energy of around 0.81 eV [3]. In addition to its excellent electrical characteristics, CGO demonstrates thermal compatibility with other SOFC components such as yttria-stabilized zirconia (YSZ) and various cathode materials, showing minimal degradation at elevated temperatures [4]. Owing to its favorable electrochemical properties, CGO enhances the electrochemical reactions at the electrode-electrolyte interface and effectively facilitates the oxidation of hydrogen and hydrocarbon fuels [5]. Its superior oxygen ion transport ability also suppresses carbon buildup at the interface, reducing coking and improving long-term performance [6]. Further enhancement in CGO’s performance has been observed through compositional modifications, particularly via nickel incorporation. Nickel-doped CGO (Ni-CGO) has demonstrated improved catalytic activity and resistance to coking, which is a common degradation issue in SOFC anodes. In addition to its high electrical and electrochemical properties, CGO exhibits reduced polarization resistance due to increased oxygen vacancy concentration, and it is less susceptible to poisoning by various fuels such as hydrogen, methane, and synthetic alternatives [7]. These attributes make CGO one of the most promising candidates for SOFC anode applications. SOFCs offer several advantages over other types of fuel cells. Their high operating temperatures (typically 600–1000°C) enable efficient fuel conversion and allow for the direct use of hydrocarbon fuels without the need for external reforming [8–10]. SOFCs also feature high electrical efficiency and do not rely on expensive catalysts such as platinum. Additionally, they are well-suited for combined heat and power (CHP) systems due to their ability to produce both electricity and high-grade heat [11,12].

Traditionally, SOFC anodes are based on nickel (Ni)-containing ceramics, such as Ni-YSZ. Nickel provides excellent electron conductivity and catalytic activity; however, challenges such as Ni coarsening and migration over time lead to a decline in electrochemical performance. Studies have shown that mechanisms beyond Ostwald ripening, such as enhanced Ni wettability and evaporation-condensation pathways, contribute to nickel redistribution within the anode structure [13]. Ni-GDC anodes present a compelling alternative to conventional Ni-YSZ systems. They offer improved catalytic behavior and reduced carbon deposition, particularly when operating on renewable fuels such as ethanol. However, long-term stability and microstructural integrity under operating conditions remain concerns. Research by Yokokawa and others has highlighted the dynamic migration of Ni and GDC phases, resulting in morphological changes that can degrade performance over time [14]. In a related context, studies on Ni-doped nanostructures for lithium-ion batteries provide insights into nickel’s influence on electrochemical behavior. For instance, Ni-doped Nb₁W₁O₉₃ nanowires exhibited excellent capacity retention (93.1% over 500 cycles at 5C) due to enhanced conductivity and active sites [15]. Similarly, Ni-doped anatase TiO₂ showed significantly improved discharge capacity (226 mAh·g⁻^1^ after 50 cycles) compared to undoped TiO₂ (132 mAh·g⁻^1^), indicating nickel’s beneficial role in enhancing structural and electrochemical performance [16]. Despite nickel’s well-known catalytic advantages, it also contributes significantly to carbon deposition, particularly at elevated temperatures. Tian Gan’s recent work demonstrated that carbon accumulation on Ni metal at 700°C led to a steady voltage drop in Ni-SDC anode-based SOFCs during a 200-minute operation, underscoring the persistent issue of coking in Ni-based systems [17].

Lanthanum strontium cobalt oxide, commonly referred to as LSCO, is one of the most sought-after cathode materials used in Solid Oxide Fuel Cells (SOFCs) due to its excellent electrochemical properties. These properties stem from its flexible stoichiometry and high oxygen reduction reaction (ORR) activity [18,19]. LSCO is a perovskite-type oxide known for its excellent electronic conductivity and catalytic activity toward oxygen reduction reactions (ORR). Its mixed ionic-electronic conducting (MIEC) properties facilitate efficient charge transfer at the cathode, which is critical for the overall performance of Solid Oxide Fuel Cells (SOFCs). Additionally, LSCO exhibits structural stability at high operating temperatures, contributing to the long-term durability of the fuel cell. The presence of cobalt ions enhances its catalytic activity for ORR, helping to reduce polarization losses at the cathode and thereby improving overall cell efficiency [20–22]. However, despite these advantages, LSCO faces challenges related to long-term operational stability. One major issue is the migration of strontium to the surface, which leads to the formation of insulating Sr-rich secondary phases. This surface segregation can block active sites for ORR, resulting in performance degradation over time. The high thermal expansion coefficient of LSCO can lead to cracking or delamination when paired with a yttria-stabilized zirconia (YSZ) electrolyte, resulting in poor long-term performance of SOFCs [23]. To address this issue, this study proposes BaNb₄MoO₂₀ (BNMO) as an alternative electrolyte material to mitigate thermal mismatch and improve structural compatibility.

BNMO is a hexagonal perovskite-related oxide that has emerged as a promising electrolyte material due to its high oxide ion conductivity and excellent redox stability. Its crystal structure facilitates efficient oxide ion transport, which is essential for the effective operation of Solid Oxide Fuel Cells (SOFCs). Furthermore, BNMO exhibits stability under both oxidizing and reducing conditions, making it well-suited for long-term SOFC applications [24]. The presence of these ions enhances both electronic and ionic conductivities, which can help reduce energy losses in SOFCs. Additionally, BNMO’s thermal expansion coefficient is well-matched with those of the anode and cathode layers, minimizing the risk of mechanical failure. This compatibility contributes to a robust mechanical structure at high operating temperatures, which is essential for maintaining the long-term structural integrity of the SOFC [25]. In this study, we report the fabrication and performance analysis of a Ni-incorporated CGO/BNMO/LSCO cell, anticipating enhanced SOFC performance. We propose that the combination of CGO as the anode, BNMO as the electrolyte, and LSCO as the cathode offers complementary properties—such as improved ionic conductivity, enhanced power density, and operational stability across a range of SOFC operating temperatures [26–28]. The combination is also expected to minimize thermal expansion mismatches, thereby ensuring the long-term mechanical integrity of the fabricated SOFC. This study presents a research strategy that integrates advanced materials science and manufacturing expertise with fundamental electrochemical analysis of individual cell components, supported by theoretical electrochemical simulations.

## 2. Materials and methods

All materials used in this study were of analytical grade. The chemicals employed in the fabrication of the solid oxide fuel cell included lanthanum nitrate hexahydrate (La(NO₃)₃·6H₂O, 99% purity), strontium nitrate (Sr(NO₃)₂, > 95% purity), gadolinium nitrate hexahydrate (Gd(NO₃)₃·6H₂O, molecular weight: 451.36 g/mol, 99.99% purity), cerium(III) nitrate hexahydrate (Ce(NO₃)₃·6H₂O, molecular weight: 434.22 g/mol, 99% purity), cobalt nitrate hexahydrate (Co(NO₃)₂·6H₂O, molecular weight: 291.03 g/mol, 98% purity), trisodium phosphate (Na₃PO₄, 99% purity), and sodium alginate (Na-ALG) with a viscosity of ≥2,000 cP (2% solution at 25°C). All chemicals were procured from Sigma-Aldrich Ltd., India.

### 2.1. Synthesis of gadolinium-doped ceria

Under magnetic stirring, 20 mL of trisodium phosphate solution (0.02 M) was gradually added dropwise to 60 mL of cerium nitrate solution (0.1 M). Gadolinium nitrate was then added to the reaction mixture in increments to achieve 8% doping, followed by vigorous stirring for 30 minutes. Continuous stirring led to the formation of a white colloid. This colloid was subsequently transferred to a 100 mL Teflon-lined autoclave and subjected to hydrothermal treatment at 180°C for 15 hours. After cooling to room temperature, the colloid was collected by centrifugation, thoroughly washed with double-distilled water and ethanol, and dried at 60°C for five hours to yield gadolinium-doped ceria (GDC) nanoparticles [29–31]. Under reducing conditions at the fuel electrode, GDC functions as a mixed ionic-electronic conductor (MIEC). It also serves as an effective catalyst for the anodic fuel oxidation reaction, which occurs across the entire GDC surface due to the mixed valence states of Ce³⁺ and Ce⁴ ⁺ . This understanding of GDC’s fundamental properties and their role in electrochemical reactions within Ni/GDC cermets suggests that finely dispersed GDC particles enhance local electron collection and ion transport. Meanwhile, the Ni phase primarily supports long-range electron transport and provides mechanical stability.

### 2.2. Synthesis of lanthanum strontium cobalt oxide

Analytical-grade metal nitrate salts of lanthanum (La(NO₃)₃·6H₂O), strontium (Sr(NO₃)₂), and cobalt (Co(NO₃)₂·6H₂O) were prepared as 0.1 M solutions in 100 mL of distilled water to serve as precursor materials. A calculated amount of citric acid was then added to the mixed solution with continuous stirring for 30 minutes. Subsequently, 2.92 g of EDTA dissolved in 100 mL of distilled water was slowly introduced into the mixture. The pH of the solution was adjusted to 0.5 using citric acid. Finally, the surfactants ethylene glycol (EG) and activated carbon (AC) were added, and the solution was continuously stirred and heated for 6 hours to form a viscous gel. The resulting gel was dried at 150°C for 12 hours, followed by drying at 250°C for 5 hours. The as-synthesized powder was then calcined at 900°C with a heating and cooling rate of 5°C/min for 5 hours [32–34].

## 3. Characterization techniques

Gadolinium-doped ceria (GDC) (Ce₀.Gd₀.₂O₂) and lanthanum strontium cobalt oxide (LSCO) (La₀.Sr₀.₄CoO₃) were characterized by Fourier transform infrared (FTIR) spectroscopy using a Perkin Elmer spectrophotometer (model 1600) with a wavelength range of 11000–30 cm⁻^1^, scanning within the wavenumber range of 400–4000 cm⁻^1^. The samples were mixed with KBr in a 1:5 ratio to prepare a uniform pellet, which was then compressed using a hydraulic press to form a 10 mm diameter plate. The crystalline structures of GDC and LSCO were analyzed by powder X-ray diffraction (XRD) using a Siemens D-5000 diffractometer with Cu Kα radiation (λ = 1.54 Å) at a scanning rate of 2° per minute. Diffraction patterns were recorded over a 2θ range of 10° to 80°. The surface morphology of GDC, BNMO, and LSCO was examined by scanning electron microscopy (SEM), with samples coated with gold via sputtering. Electrochemical impedance spectroscopy (EIS) measurements were performed using a CH Instruments 660D electrochemical workstation (CH Instruments, Inc., USA).

## 4. Results and discussion

### 4.1. FTIR spectra

**Fig 1:**
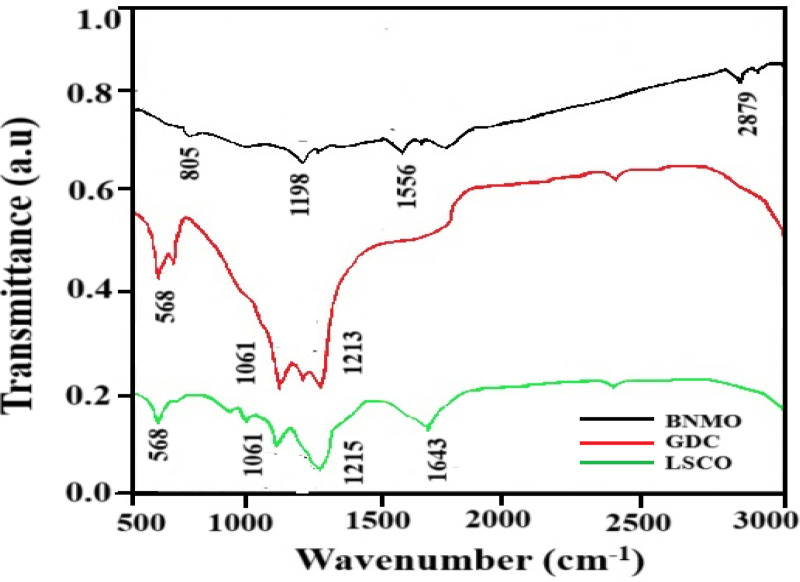
FTIR spectra of (a) Ba_7_Nb_4_MoO_20_ electrolyte (b) Gadolinium-doped ceria and (c) Lanthanum strontium cobalt oxide.

Fig 1 shows the FTIR spectra of (a) BaNb₄MoO₂₀ electrolyte, (b) gadolinium-doped ceria (GDC), and (c) lanthanum strontium cobalt oxide (LSCO). Fourier-transform infrared spectroscopy (FTIR) is a powerful, non-destructive technique for analyzing SOFC materials and performance. It enables the investigation of chemical processes within the fuel cell, providing insights into reaction mechanisms and material interactions essential for optimizing cell efficiency.

Fig 1(a) displays the FTIR spectrum of the BaNb₄MoO₂₀ electrolyte, showing characteristic peaks at 805 cm⁻^1^, attributed to Ba-O stretching vibrations; 1198 cm⁻^1^, corresponding to the Mo-O vibrational mode at the tetrahedral site; 1556 cm⁻^1^, assigned to Ba-O-Nb in-plane stretching; and 2879 cm⁻^1^, related to O-H stretching and bending vibrations [35].

Fig 1(b) presents the FTIR spectrum of Ce₀.Gd₀.₂O₂ (GDC), with key peaks at 568 cm⁻^1^ corresponding to Ce-O stretching at the tetrahedral site; 1061 cm⁻^1^ for Gd-O stretching at the octahedral site; and 1213 cm⁻^1^ attributed to the symmetric vibrational mode of ʋ-COO⁻ in-plane [36].

Fig 1(c) shows the FTIR spectrum of La₀.Sr₀.₄CoO₃ (LSCO), featuring characteristic peaks at 568 cm⁻^1^ corresponding to Sr-O stretching in-plane; 1061 cm⁻^1^ for Co-O stretching at the octahedral site; 1215 cm⁻^1^ for the symmetric vibrational mode of ʋ-COO⁻ in-plane; 1643 cm⁻^1^ assigned to the La-O-Co symmetric vibrational mode in-plane; and 2456 cm⁻^1^ related to O-H stretching and bending vibrations [37–40].

### 4.2. XRD pattern

Nanomaterials have garnered significant attention in recent years due to their unique physical and chemical properties, which arise from their nanoscale dimensions. One of the key techniques for characterizing these materials is X-ray diffraction (XRD). XRD is a powerful analytical tool that allows researchers to determine the crystalline structure, phase composition, and crystallite size of nanomaterials.

**Fig 2:**
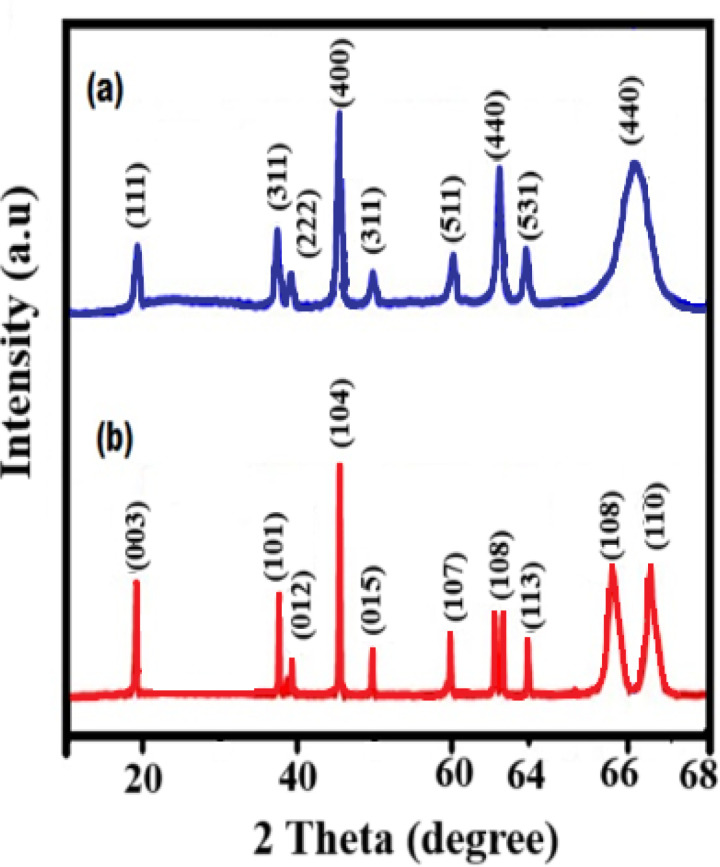
XRD pattern of (a) Gadolinium-doped ceria and (b) Lanthanum strontium cobalt oxide.

Fig 2(a) shows the XRD pattern of gadolinium-doped ceria (GDC), with characteristic peaks at 2θ values of 19.8°, 38.7°, 39.4°, 46.8°, 50.2°, 61.2°, 62.8°, 64.1°, and 66.2°, corresponding to the (111), (311), (222), (400), (311), (511), (440) planes, respectively (JCPDS: 048–0124), confirming the formation of a fluorite structure. The crystallite size of the Ce₀.Gd₀.₂O₂ nanoparticles was estimated to be 7.2 nm.

Fig 2(b) shows the XRD pattern of lanthanum strontium cobalt oxide (LSCO), with characteristic peaks at 2θ values of 24.6°, 33.8°, 38.6°, 39.2°, 42.1°, 48.2°, 59.3°, 62.8°, 66.2°, and 67.2°, corresponding to the (003), (101), (012), (104), (015), (107), (108), (110), (113), and (110) planes, respectively (JCPDS: 043–0161), confirming the formation of a cubic perovskite structure [41, 42]. The crystallite size of the LSCO nanoparticles was found to be 5.4 nm.

### 4.3. SEM analysis

**Fig 3:**
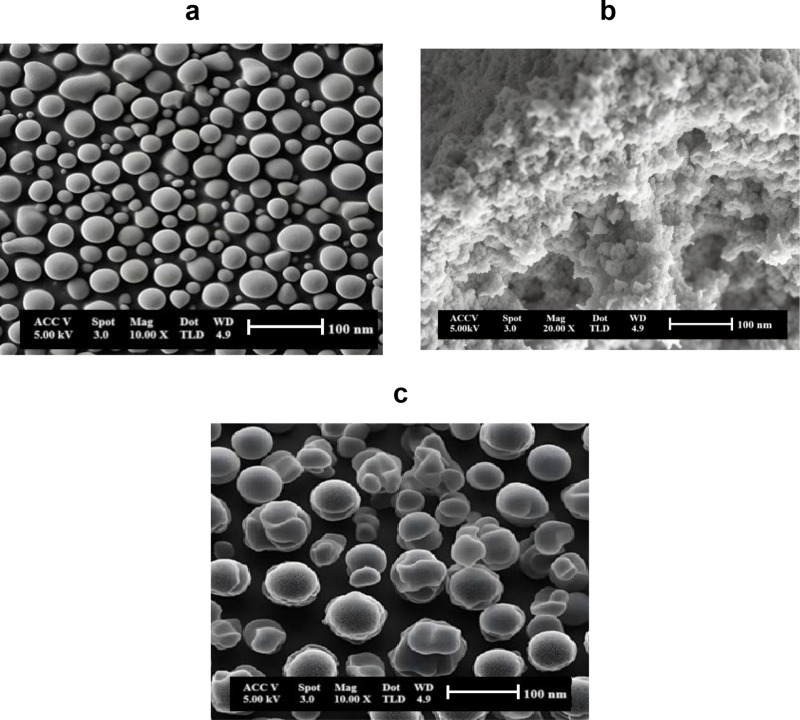
SEM image of (a) Lanthanum strontium cobalt oxide (b) Ba_7_Nb_4_MoO_20_ electrolyte and (c) Gadolinium-doped ceria.

Fig 3 shows SEM images of (a) lanthanum strontium cobalt oxide (LSCO), (b) BaNb₄MoO₂₀ (BNMO) electrolyte, and (c) gadolinium-doped ceria (GDC). Surface morphology plays a crucial role in SOFC performance because it directly affects electrochemical reactions. The interfaces of the anode and cathode are sites of critical reactions; therefore, an optimized surface morphology can significantly enhance reaction rates by providing more active sites for electrochemical processes. For instance, nanostructured materials or porous designs improve mass transport phenomena while increasing the effective surface area available for reactions, leading to higher overall cell efficiency and reduced polarization losses during operation [43].

Fig 3(a) shows the SEM image of LSCO, revealing a homogeneous, regularly sized granular structure with particles approximately 54 nm in diameter. The electrochemical activity of the cathode is closely related to its surface morphology, as a larger surface area promotes faster conversion of reactants into energy. The high surface area of LSCO thus makes it a promising cathode material for SOFCs.

Fig 3(b) presents the SEM image of the BNMO electrolyte, which exhibits a well-defined surface with high porosity. This porosity facilitates enhanced ion conduction and gas diffusion, thereby improving reaction rates. Furthermore, specific morphological features can promote favorable catalytic behavior at electrode interfaces.

Fig 3(c) shows the SEM image of GDC, displaying a homogeneous granular structure with particle sizes around 63 nm [44].

### 4.4. DC conductivity

**Fig 4:**
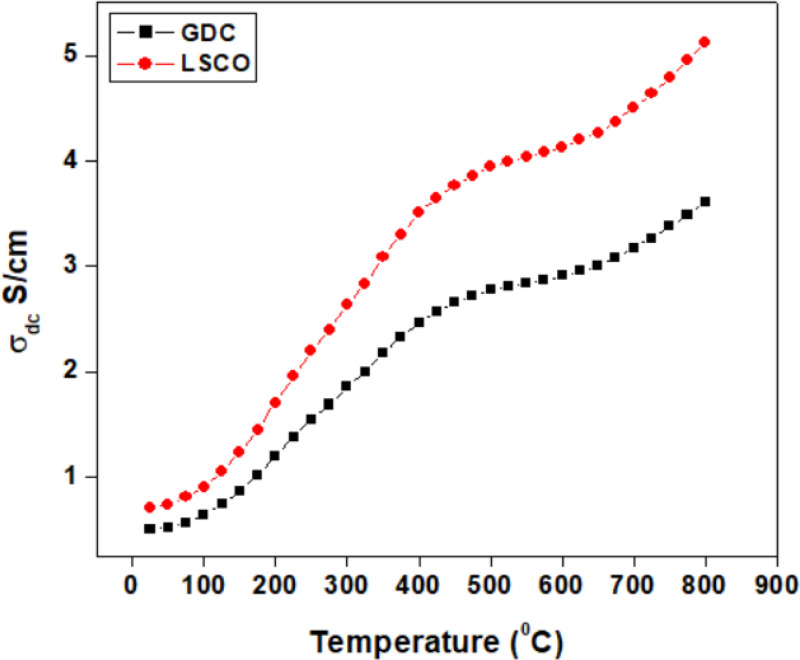
DC conductivity of (a) Gadolinium-doped ceria and (b) Lanthanum strontium cobalt oxide.

Fig 4 shows the DC conductivity of (a) lanthanum strontium cobalt oxide (LSCO) and (b) gadolinium-doped ceria (GDC) as a function of temperature up to 800°C. It is observed that the conductivity of both GDC and LSCO nanoparticles increases with rising temperature. Ionic conductivity generally increases with temperature due to the enhanced mobility of ions within the material. As thermal energy rises, the vibrational motion of lattice atoms intensifies, reducing resistance to ion migration. This effect is especially pronounced in solid electrolytes and molten salts, where higher temperatures facilitate the dissociation of ionic compounds into charged species, thereby enhancing conductivity.

At lower temperatures (25°C to 300°C), conductivity increases gradually due to improved ionic mobility through intrinsic and oxygen vacancies. At higher temperatures (300°C to 800°C), the increase in conductivity is primarily attributed to the hopping of charge carriers from lower to higher energy states after absorbing thermal energy [45,46]. Notably, LSCO exhibits a high conductivity of 5.2 S/cm compared to GDC, making it a suitable cathode material for SOFCs.

The temperature-dependent ionic conductivity has significant implications for applications such as batteries and fuel cells. Elevated temperatures improve the rate of oxygen ion transport between electrodes during charge and discharge cycles. However, this relationship is not strictly linear, as excessively high temperatures may cause adverse effects like increased side reactions or material degradation [47].

### 4.5. Electrochemical Impedance Spectroscopy (EIS)

Electrochemical Impedance Spectroscopy (EIS) measurements are typically conducted at the operating temperature of Solid Oxide Fuel Cells (SOFCs), around 850°C, to ensure relevance to real-world conditions. The anode side is supplied with a reducing gas mixture (e.g., H₂/N₂), while the cathode side receives an oxidizing gas. A small-amplitude sinusoidal AC voltage perturbation, usually between 10 and 30 mV, is applied to maintain system linearity and avoid disturbances. The frequency spectrum ranges from high frequencies down to 0.1 Hz, capturing processes occurring across different time scales. A potentiostat/galvanostat with a built-in impedance analyzer was used to record the spectra. The results are typically plotted as Nyquist or Bode plots to visualize and analyze the electrochemical processes.

**Fig 5:**
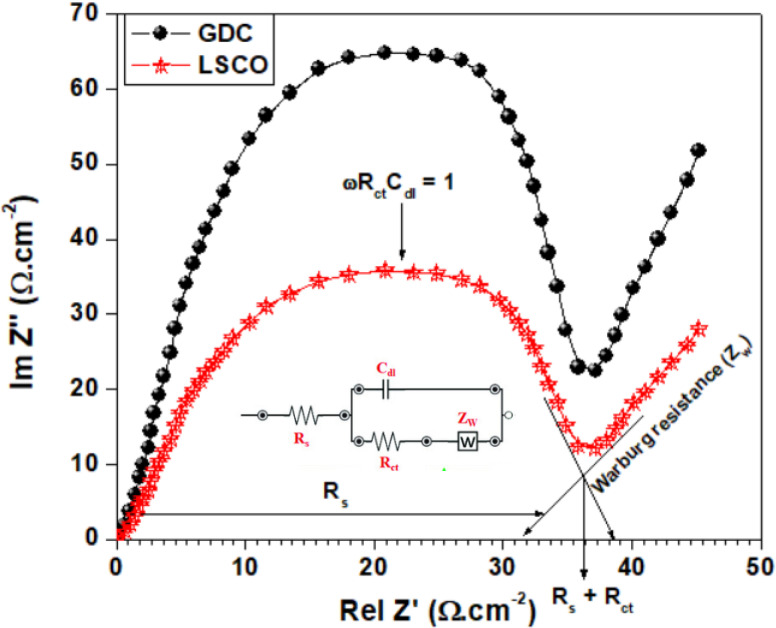
EIS impedance of (a) Gadolinium-doped ceria and (b) Lanthanum strontium cobalt oxide.

Fig 5 shows the EIS impedance spectra plotted in Nyquist coordinates for (a) gadolinium-doped ceria and (b) lanthanum strontium cobalt oxide. Nyquist plots reveal key characteristics of electrochemical systems, such as charge transfer resistance and double-layer capacitance. The semicircular arcs observed in these plots correspond to specific physical phenomena within the system. For example, the diameter of each arc represents the charge transfer resistance, often associated with grain boundary resistance. The initial resistance at the high-frequency intercept corresponds to the bulk properties of the electrolyte, while the increase in resistance following the first semicircle is attributed to diffusion resistance at lower frequencies, reflecting electrode polarization [48].

Charge transfer processes are influenced by different oxygen reduction and hydrogen oxidation reactions, as well as the distinct characteristics of the rate-determining steps in these electrode reactions. The oxygen reduction process is often limited by the slow interfacial exchange of oxygen with the gas phase. In both air and wet hydrogen atmospheres, the polarization resistance of the LSCO electrode decreases as the strontium content in the supporting electrolyte increases – a significant observation from these data [49].

S. G. Arbati et al. [50] reported that the overall performance and electrical properties of anode materials strongly correlate with their microstructure, as observed in the Nyquist plots. According to their findings, the electrochemical activity of NiO/GDC (NGC) cermet anodes for the H₂ oxidation reaction at the electrode-electrolyte interface is enhanced through appropriate structural modifications. NiO/GDC cermet anodes exhibit higher activity when optimized for enhanced porosity, increased active sites at the triple-phase boundary (TPB), and smaller particle sizes. At 800°C, the half-cell with NGC anode powder demonstrated the lowest polarization resistance (0.106 Ω·cm^2^). Additionally, the presence of nickel-doped GDC in the anode resulted in a low polarization resistance of 0.82 Ω·cm^2^. Nyquist plots can inherently accommodate multiple time constants characteristic of complex systems [50]. In many cases involving porous electrodes or heterogeneous reactions, multiple semicircles may appear, each corresponding to different mechanistic pathways. This feature allows researchers to effectively disentangle overlapping processes.

A commonly used model for interpreting EIS data is the Randles circuit, which effectively represents the electrochemical processes occurring at the electrode-electrolyte interface. This circuit typically includes the solution resistance (Rs), which accounts for the resistance of the electrolyte and any inherent resistances within the system. The charge transfer resistance (Rct) corresponds to the resistance against charge transfer across the electrode-electrolyte interface, while the double-layer capacitance (Cdl) models the capacitive behavior resulting from the formation of the electric double layer at the interface. In systems where diffusion processes are significant, a Warburg impedance (Zw) element is included to account for mass transport limitations. The Warburg element is characterized by a 45° line in the low-frequency region of the Nyquist plot, indicating diffusion-controlled processes [51].

### 4.6. SOFC testing

**Fig 6:**
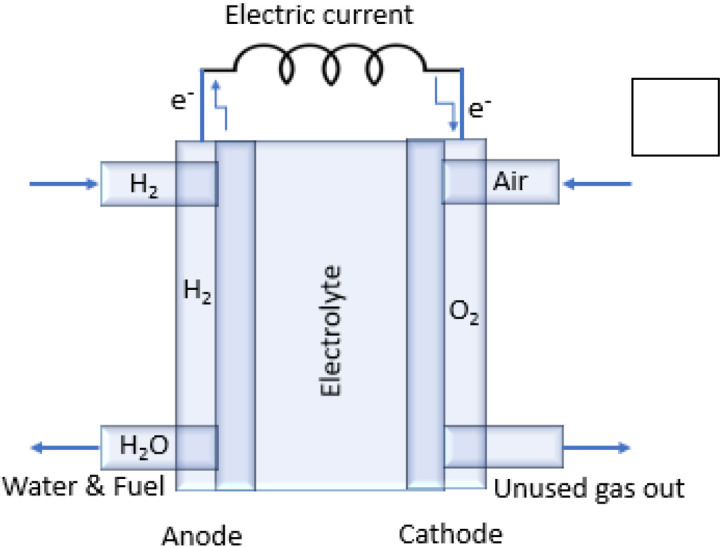
Fabrication of (a) schematic diagram of solid oxide fuel cell using GDC/BNMO/LSCO and (b) shows the cell components that serve as structural materials.

The experimental setup consists of a furnace, a gas supply system, a humidifier, current collectors, sealing materials, a data acquisition system, and control software. A high-temperature furnace capable of maintaining stable temperatures up to 1700°C was utilized for solid oxide fuel cell (SOFC) testing. Mass flow controllers regulate the flow rates of fuel and oxidant gases to the anode and cathode, respectively, while a humidifier adjusts the humidity levels in the testing chamber. Platinum meshes or wires are attached to the electrodes to serve as current collectors. High temperature sealing materials are employed to prevent gas leakage and ensure separation between the anode and cathode compartments [52]. A data acquisition system records voltage, current, and impedance data in real-time, allowing continuous monitoring of the SOFC’s performance. An illustration of the test stand, gas flow paths, and electrical connections is provided in Fig 6(a), offering a visualization of the testing environment.

Typically, SOFCs include cell components that serve as structural materials to support the other layers, with diameters less than 10 mm. The main components of SOFCs are the electrolyte, anode, and cathode. For this study, the materials used are as follows: BaNb₄MoO₂₀ as the electrolyte; Ce₀.Gd₀.₂O₂ (GDC) as the anode material, prepared by the sol-gel method, where the metallic part is typically nickel combined with GDC, as shown in Fig 6(b); and LSCO (La₀.Sr₀.₂CoO₃) as the cathode material [53–55]. The anode is typically a porous composite coated over nickel. Higher porosity and lower tortuosity indicate better gas permeability; however, it is important to balance mechanical integrity and resistance to thermal stresses with high pore volume and low tortuosity. Gas permeability increases past a 30% percolation limit in total pore volume, reaching a saturation value of approximately 50% before the thin layer deposition of the cathode and electrolyte [56,57]. For electrode deposition, a suspension was prepared using LSCO powders in an isopropanol-ethanol mixture (1:3 ratio), with polyvinyl butyral (PVB) as a binder and Beycostat as a dispersant agent. In a conventional anode-supported SOFC, the electrolyte was deposited using wet powder spraying (WPS) and annealed in a reducing atmosphere of N₂ at 550°C with a heating rate of 1°C/min. The anode and electrolyte were co-sintered at 1100°C in air. Subsequently, the LSCO cathode was dip-coated and sintered in air at 1150°C. Highly reactive layers composed of fine particles are essential for improving the sintering process; however, extremely reactive particles tend to agglomerate, resulting in porous sintered structures. To mitigate this, stable and concentrated colloidal suspensions must be prepared, using dispersants to control interparticle forces [58].

**Fig 7:**
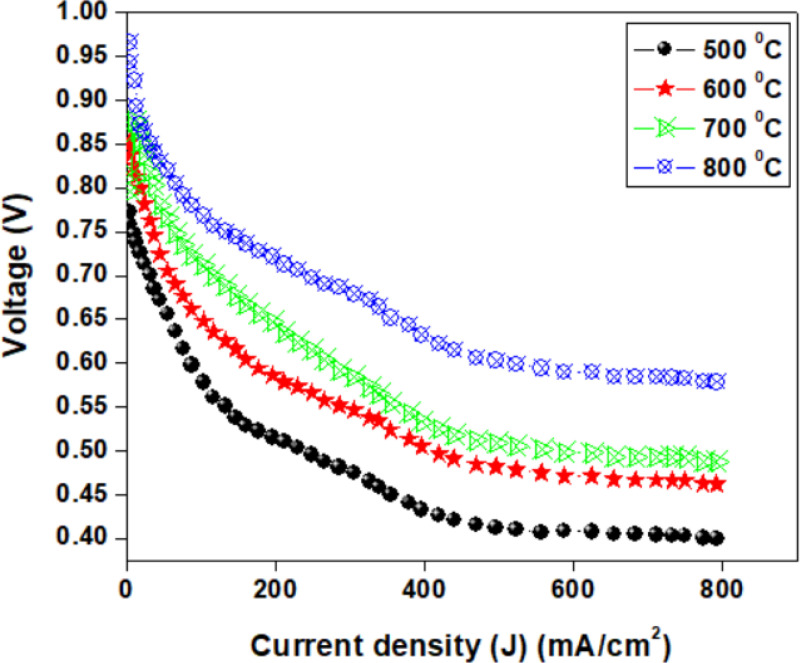
The variation of voltages against different current density.

**Fig 8:**
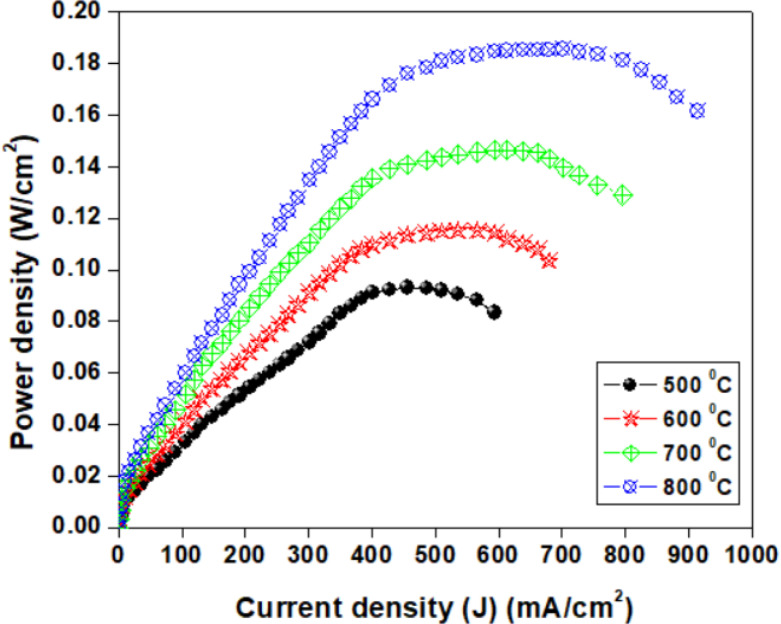
The variation of power density against current.

Figs 7 and 8 display the current-voltage (I-V) and current-power (I-P) curves of the fuel cells under investigation, with the anode supplied with wet hydrogen and the cathode with ambient air. Bruno L. Augusto et al. [59] studied two cell configurations: (i) a fuel cell with a catalytic layer (cathode/electrolyte/anode/catalyst), in which a low Ni-content (18 wt.%) Ni/GDC cermet was deposited onto a Ni/YSZ anode; and (ii) a fuel cell with a catalytic layer (cathode/electrolyte/anode/catalyst). To assess the long-term stability of the latter SOFC, it was operated continuously at 1123 K for approximately 50 hours. Fig 7 shows the I-V characteristics of an SOFC, illustrating the relationship between the direct current (DC) flowing through the cell and the DC voltage across its terminals. The voltage plotted as a function of current density reflects contributions from various fundamental processes to the voltage drop, as determined by SOFC-EIS measurements [60]. The slope of the voltage drop due to membrane resistance is about half of the overall slope of the I-V curve in the linear voltage range. Therefore, membrane resistance accounts for approximately 50% of the total voltage loss, with the remainder attributed to charge transfer resistance, which increases continuously even at high current densities [61]. It is also noted that the voltage dropped stepwise when the current density reached 200 mA/cm^2^, decreasing from 0.75 V to 0.53 V at 500°C and from 0.98 V to 0.73 V at 800°C, possibly due to charge transfer resistance at the composite interface.

Kazuhiro Yamamoto et al. [62] fabricated a high-performance Ni-GDC-nanocube anode for low-temperature SOFCs using a unique solution-based chemical reaction method. The anode material was composed of metallic Ni spheres covered with highly reactive GDC nanocubes, forming a core-shell structure. Without any sintering, this distinctive composite structure introduced a substantially longer electrical path (>20 μm). The metallic Ni spheres crystallized at temperatures as low as 500°C during power-generation tests, creating an effective framework. This structure demonstrated an extremely efficient electrical pathway from the electrolyte-anode interface to the anode’s edge (electrical collection point), as reflected by the high power density [63]. A power-generation test was conducted at 600°C with a 2-μm-thick anode to verify this hypothesis. Our achievement of a nearly identical power density compared to a 20-μm-thick anode indirectly confirms that the oxygen diffusion distance was relatively short, less than 2 μm. Fig 8 shows that the electrolyte-supported BNMO cell exhibited good performance. At 500°C, 600°C, 700°C, and 800°C, the cell power density reached approximately 0.094, 0.118, 0.146, and 0.184 W/cm^2^, respectively, which is a strong result for a fuel cell with a supporting electrolyte. The performance of the fuel cell with the BNMO electrolyte was slightly lower due to the reduced conductivity of the electrolyte without Mo and decreased electrochemical activity of the electrodes in contact with the BNMO electrolyte [64]. The electrochemical activity of LSCO electrodes is known to be lower, particularly in oxidizing atmospheres, due to significant electrode polarization. This makes it challenging to assess the impact of the supporting electrolyte on cell performance. To enhance power characteristics and reduce polarization resistance, the electrodes were impregnated with a 10 ml saturated solution of Sr(NO₃)₂, which has previously been shown to be an effective electrocatalyst for symmetrical electrodes [65]. As temperature decreases, the difference in power density between the original and impregnated electrodes becomes negligible, as overall voltage reduction is more significantly attributed to the cell’s ohmic resistance rather than electrode polarization. Future research will focus on increasing specific conductivity and decreasing the thickness of the supporting electrolyte to improve cell efficiency at lower temperatures.

## 5. Conclusions

The solid oxide fuel cell (SOFC) was fabricated using nanocomposites such as Gadolinium-doped ceria (GDC) and Lanthanum strontium cobalt oxide (LSCO), both prepared using the sol-gel method. The nanocomposites were characterized using FTIR, XRD, and SEM to analyze their structural properties and surface morphology. FTIR spectra confirmed the presence of ceria in the GDC nanocomposites and strontium in the LSCO material.

The XRD spectra of GDC showed characteristic peaks at specific 2θ values, confirming the formation of a fluorite-type structure with an estimated crystallite size of approximately 7.2 nm. For LSCO, the XRD patterns confirmed the formation of a cubic perovskite structure with a crystallite size of about 5.4 nm. SEM analysis revealed that the surface morphology of both LSCO and GDC consisted of homogeneously distributed, regularly sized granular structures, with average particle sizes of 54 nm and 63 nm, respectively.

Thermal conductivity studies indicated that, between 25°C and 300°C, conductivity increased gradually due to enhanced ionic mobility through ionic channels and oxygen vacancies. At elevated temperatures (300°C to 800°C), conductivity improved further, likely due to charge carrier hopping from lower to higher energy states facilitated by absorbed thermal energy. Notably, LSCO exhibited a high conductivity of 5.2 S/cm, outperforming GDC and confirming its suitability as a cathodic material for SOFCs.

The power density curve demonstrated that the fuel cell achieved power densities of approximately 0.094, 0.118, 0.146, and 0.184 W/cm^2^ at 500°C, 600°C, 700°C, and 800°C, respectively, indicating strong performance of the SOFC utilizing the BNMO supporting electrolyte.
